# Effect of Fish Oil Supplementation on Hepatic and Visceral Fat in Overweight Men: A Randomized Controlled Trial

**DOI:** 10.3390/nu11020475

**Published:** 2019-02-23

**Authors:** Helen M. Parker, Jeffrey S. Cohn, Helen T. O’Connor, Manohar L. Garg, Ian D. Caterson, Jacob George, Nathan A. Johnson

**Affiliations:** 1 Faculty of Health Sciences, The University of Sydney, Lidcombe, NSW 2141, Australia; helen.oconnor@sydney.edu.au (H.T.O.); nathan.johnson@sydney.edu.au (N.A.J.); 2 Charles Perkins Centre, The University of Sydney, Camperdown, NSW 2006, Australia; 3 Nutrition & Metabolism Group, The Heart Research Institute, Newtown, NSW 2042, Australia; cohnj@hri.org.au; 4 School of Biomedical Sciences & Pharmacy, Faculty of Health and Medicine, University of Newcastle, Callaghan, NSW 2308, Australia; manohar.garg@newcastle.edu.au; 5 Boden Institute of Obesity, Nutrition, Exercise and Eating Disorders, The University of Sydney, Camperdown, NSW 2006, Australia; ian.caterson@sydney.edu.au; 6 Storr Liver Centre, The Westmead Institute for Medical Research, The University of Sydney, Westmead, NSW 2145, Australia; jacob.george@sydney.edu.au

**Keywords:** omega-3 PUFA, polyunsaturated fatty acids, RCT, non-alcoholic fatty liver, body composition

## Abstract

Being overweight increases the risk of the development of metabolic conditions such as non-alcoholic fatty liver disease (NAFLD), which is itself an independent predictor of cardiovascular disease. Omega-3 polyunsaturated fatty acid (PUFA) supplementation is recommended for prevention of chronic disease, and is thought to reduce raised liver fat, yet there have been few randomized controlled trials with accurate measurement of liver fat. We assessed the effect of 12 weeks of supplementation with omega-3 PUFA from fish oil versus placebo on quantified liver fat, liver tests, and body composition including visceral adipose tissue (VAT) in a double-blind randomized controlled trial. Fifty apparently healthy overweight men (BMI 25.0–29.9 kg/m^2^; waist > 94 cm) were randomly allocated to consume fish oil (total daily dose: 1728 mg marine triglycerides, of which 588 mg EPA and 412 mg DHA, combined with 200 mg antioxidant, coenzyme Q10) or placebo (olive oil capsules) daily for 12 weeks. Liver fat was assessed using proton magnetic resonance spectroscopy. All outcomes were assessed at baseline and following 6 and 12 weeks of supplementation. Baseline liver fat was 4.6 ± 0.5% (range: 0.6 to 18.2%); 16 (32%) participants met the criteria for NAFLD (>5.5% liver fat). Repeated measures ANOVA revealed no significant time or group × time effect for fish oil versus placebo for liver fat, liver enzymes, anthropometry, or body composition including VAT (*p* > 0.05 for all), with similar finding for sub-analysis of participants with NAFLD. Omega-3 PUFA did not appear to be an effective agent for reducing liver fat in overweight men. The factors determining the health benefits of omega-3 PUFA supplementation on an individual level need to be clarified.

## 1. Introduction

Despite the well-recognized health consequences of excess body fat, epidemiological data show that the worldwide prevalence of overweight/obesity is continuing to increase [1]. Increased visceral fat in particular, predisposes individuals to type 2 diabetes, hypertension, and dyslipidemia [2]. Non-alcoholic fatty disease (NAFLD), an independent risk factor for cardiovascular disease (CVD) [3], is also positively associated with increased adiposity, being present in around 20–40% of the general population, and up to 90% of obese individuals [4].

Systematic reviews of treatments for NAFLD indicate there are few effective pharmacological agents for reducing liver fat, and these often also result in adverse effects such as weight gain [5,6]. Diet-induced weight loss of as little as 3% is effective in reducing both visceral and hepatic adipose tissue [7], and such changes are associated with a reduction in traditional cardiovascular risk factors [7,8]. In the absence of other suitable therapies, weight loss by energy restriction and exercise remains the first line of treatment for reducing visceral obesity and hepatic steatosis [6].

However, evidence from randomized controlled trials clearly shows that for most patients, weight loss is difficult to sustain [9]. Consequently, research has focused on the efficacy of other non-pharmacological approaches which target liver fat reduction. Nutraceuticals, or nutrients used in a manner similar to pharmaceuticals, represent an area of growing interest, and although data remain scarce, the efficacy of several nutraceuticals for NAFLD management has been examined [6].

Omega-3 polyunsaturated fatty acids (PUFA) are essential nutrients not synthesized in the human body and therefore must be obtained from the diet [10]. Fish and seafood constitute the richest dietary sources of eicosapentaenoic acid (EPA) and docosahexaenoic acid (DHA), the forms of omega-3 PUFA associated with the greatest health benefits [10,11]. Given that omega-3 PUFA are found in significant quantities in a limited number of foods, the average individual habitual intake of omega-3 PUFA can vary considerably in the Western diet [12]. A low dietary intake of omega-3 PUFA is associated with increased hepatic fat [13], and total parenteral nutrition studies have confirmed that chronic dietary omega-3 PUFA deficiency leads to hepatic steatosis [14,15,16].

Evidence from animal [14,17] and human [18] research suggests that dietary supplementation with omega-3 PUFA may prevent NAFLD or reduce liver fat, and that this may occur independently of weight loss [14,17,18]. Furthermore, omega-3 PUFA may augment the reduction in whole body fat seen during diet-induced weight loss [19], however evidence is lacking as to whether omega-3 PUFA can elicit this effect on visceral or subcutaneous adipose tissue levels without a traditional dietary/exercise induced weight loss program. Reviews over the past few years have continued to build on early evidence for the benefit of omega-3 on liver fat [18,20], and have started to look more closely at the different presentations of fatty liver, including biopsy-proven non-alcoholic steatohepatitis (NASH) [21], and disease in both children and adults [22,23]. A common feature outlined in these reviews is a lack of assessment of liver fat quantity, with few reporting any quantitative assessment of liver fat itself (such as histologically- or proton magnetic resonance spectroscopy (^1^H-MRS)-quantified liver fat), instead relying on surrogate markers or risk factors such as liver enzymes and blood lipids. These studies clearly demonstrate the need for more evidence from randomized controlled trials with quantification of the effects on liver fat before application in clinical practice. Furthermore, most human trials assessing the effect of omega-3 PUFA supplementation on liver fat to date have employed co-treatment with diet and/or lifestyle interventions that might confound the independent effect of the nutraceutical [18].

As raised liver fat and visceral fat are likely to be present in overweight individuals, and as these represent modifiable risk factors for cardiovascular disease, the aim of this study was to assess the effect of dietary supplementation with omega-3 PUFA on liver, visceral, and abdominal subcutaneous adipose tissue in overweight men using gold-standard approaches, independent of diet and/or lifestyle interventions. The supplement used in the present study contained fish oil combined with antioxidant coenzyme Q10, which was intended to protect the unsaturated omega-3 PUFA from oxidation [24] at a dose not believed to have a biological effect in humans [25,26]. Our hypothesis was that, compared to olive oil placebo, 12 weeks of supplementation with fish oil and coenzyme Q10 would result in a reduction in liver fat in overweight men. However, while omega-3 PUFA is understood to influence lipid oxidation in vivo and hence may alter body composition, as participants were not instructed to change body weight over the study period, we hypothesized that supplementation would not result in an associated change in visceral or abdominal subcutaneous adipose tissue.

## 2. Materials and Methods

### 2.1. Participants

Fifty overweight men were recruited from the general community (Sydney, Australia) between 2011 and 2013. To be eligible, participants were non-smokers between 18 to 60 years, with a body mass index (BMI) between 25.0 and 29.9 kg/m^2^ and waist circumference >94 cm (the Caucasian cut-off values for increased CVD risk). As this research was funded by a company that supplied nutritional supplements for healthy individuals, limitations set by the Therapeutic Goods Administration (Australia) required exclusion of individuals with a clinical condition (for example, obesity, dyslipidemia, diabetes mellitus), and as such the inclusion criteria were designed to capture apparently healthy individuals with risk factors for chronic disease, rather than individuals with diagnosed chronic disease. Consistent with the majority of studies in NAFLD and omega-3, volunteers were excluded if they had uncontrolled hypertriglyceridemia, renal disease, had been previously diagnosed with diabetes mellitus, liver disease, were taking lipid-lowering medication, or if they reported taking supplements containing omega-3 PUFA within the previous 6 months. Other exclusion criteria included reporting regular alcohol intake exceeding two standard drinks (20 g ethanol) per day, or reporting recent (within 3 months) changes to diet and exercise habits or body weight (≥5% weight change).

This study was conducted according to the guidelines laid down in the Declaration of Helsinki and all procedures involving human subjects were approved by the Sydney Local Health District Ethics Review Committee (RPAH Zone, protocol number X10-0115), and registered with the Australia and New Zealand Clinical Trials Registry, ACTR number ACTRN12610000351011, for which baseline data were partially reported previously as part of a larger investigation [27]. Written informed consent was obtained from all participants prior to participating in the study.

### 2.2. Experimental Design

The study was a 12-week randomized double-blind placebo-controlled trial. Participants were randomized to either the Fish oil or Placebo arm (*n* = 25 per group). Randomization was performed by a block randomization schedule provided by an external randomization service. One researcher (HP) screened and enrolled participants, and the random allocation sequence was implemented via sequentially numbered but otherwise identical sealed capsule containers allocated to participants in the order or enrolment. The randomization code was provided in sealed envelopes only to be broken at the end of the clinical trial (following the analysis of all liver fat and abdominal fat images, and prior to statistical analysis) or in the case of serious adverse event. The Fish Oil participants consumes 4 × 500 mg capsules (total dose: 1728 mg fish oil per day, including 588 mg EPA and 412 mg DHA), with 200 mg antioxidant (coenzyme Q10) present in the capsule contents (50 mg coenzyme Q10 per 500 mg capsule) to protect the highly unsaturated fatty acids from oxidation [26] (Blackmores Pty Ltd., Warriewood, NSW, Australia). This dose of omega-3 fatty acids was chosen as it is consistent with the current recommendations for secondary prevention of CVD [28], and is a dose commonly used in studies of NAFLD [18]. While coenzyme Q10 was included in the fish oil capsules to protect the highly unsaturated fatty acids from oxidation [26], it was not included in the olive oil capsules as monounsaturated fatty acids in olive oil are expected to be more resistant to oxidation, and the dose of coenzyme Q10 present was not expected to result in health benefits for the Fish Oil participants [25,26]. Individuals in the Placebo group each consumed 4 × 500 mg capsules containing olive oil (Blackmores Pty Ltd., Warriewood, NSW, Australia). Compliance was calculated as follows: total number of capsules taken ÷ total number capsules prescribed × 100. Capsule contents were masked by gel coating and the inclusion of a “musk” scent in the capsule containers. Compliance to treatment allocation was confirmed via change in the Omega-3 Index [29]. To check the adequacy of the blinding, at each return visit following initiation of supplementation, participants were asked to identify whether or not they were on the Fish Oil or Olive Oil Placebo supplements. Measurements (see below) were undertaken at a tertiary research facility at baseline, 6, and 12 weeks.

### 2.3. Measurements

#### 2.3.1. Anthropometric Assessment

Measurements were taken in light clothing and no shoes in a University office fitted out for individual health consults including blood collection. Standing height was taken to the nearest 0.5 cm. Weight was measured using a digital platform scale accurate to 0.1kg (Tanita BC-418 Body Composition Analyzer, Tanita Corporation, Tokyo, Japan). This digital platform scale also estimated body fat (percent, mass) using bioelectrical impedance analysis (BIA). Waist circumference was measured to the nearest 0.1 cm at the midpoint between then 12th rib and the iliac crest, according to World Health Organization guidelines [30].

#### 2.3.2. Biochemical Parameters

Following an overnight (>8 h) fast, a 5 mL venous blood sample was collected from the antecubital vein. Biochemical analyses were performed by the Royal Prince Alfred Hospital Pathology Laboratory. Serum aminotransferases (ALT, AST, GGT) and triglycerides (TG) were analyzed by photometric reactions (C702 Cobas8000 Autoanalyzer, Roche Diagnostics, Indianapolis, IN, USA). Red blood cells were separated from plasma and used for Omega-3 Index testing. The Omega-3 Index was calculated as the erythrocyte membrane content of eicosapentaenoic acid (%EPA) plus docosahexaenoic acid (%DHA) expressed as a percentage of total erythrocyte membrane fatty acids, and was measured via direct transesterification of the washed erythrocytes followed by gas chromatography; methods as reported elsewhere [27,31].

#### 2.3.3. Habitual Dietary and Physical Activity Control

Participants were asked to maintain their habitual activity and eating behaviors for the duration of the intervention. Mean duration of sedentary time, physical activity, steps per day and daily energy expenditure were measured using a tri-axial accelerometer worn on the upper arm. This also estimated energy expenditure through galvanic skin responses and heat flux (SenseWear^TM^ BodyMedia Inc., Pittsburgh, PA, USA) and was measured for three days (two weekdays and one weekend day) at baseline, and during weeks 6 and 12. Accelerometers were worn 24 h per day except during water-based activities such as showering. Participants completed a three-day food record (2 weekdays and one weekend day) using household metric measures, and a validated physical activity questionnaire [32] during the same three-day period. Food records were validated by interview and analyzed by a dietitian blinded to group allocation. Average daily intakes of energy and macronutrients were quantified by dietary analysis software (Foodworks^TM^ 7 Professional Edition v7.0.3016, Xyris Software, Brisbane, QLD, Australia). Accelerometer data were analyzed by an assessor blinded to group allocation and values were averaged over 24 h. Data were omitted from analysis if the monitor was worn for <90% of a 24 h period.

#### 2.3.4. Magnetic Resonance Imaging (MRI) and Proton Magnetic Resonance Spectroscopy (^1^H-MRS)

MRI and ^1^H-MRS methods for quantifying abdominal visceral (VAT) and subcutaneous (SAT) adipose tissue, and liver fat (intrahepatic lipid; IHL) concentration and composition, respectively, are as reported elsewhere [27,33]. Briefly, a 1.5 Tesla Achieva whole-body system (Philips Medical Systems, North Ryde, NSW, Australia) was used for all MRI and ^1^H-MRS data acquisition, with participants lying supine for all measurements. MRI was used for SAT and VAT measurement, with images captured during suspended end-expiration, with 10 mm slice thickness and interslice gap of 10 mm. Images were processed using automated software (HippoFat^TM^ version 2.11, CNR Institute of Clinical Physiology, Pisa, Tuscany, Italy) [34] to calculate volume of SAT and VAT. IHL concentration and composition were measured via 1H-MRS using the whole-body (Q body) (transmit) coil and a circular polarized surface (flex M multi-channel surface) (receive) coil, with volumes of interest centered within the right lobe of the liver, and analyzed for IHL and hepatic fat composition using magnetic resonance user interface software (jMRUI version 3.0, EU Project) as described elsewhere [33,35]. Participants were subsequently grouped as those with NAFLD (IHL ≥ 5.5%) and without NAFLD (IHL < 5.5%) [36].

To ensure consistency across subjects all MRI and ^1^H-MRS analyses were performed by a single investigator blinded to treatment allocation and data collection time point.

### 2.4. Statistical Analysis

The primary outcome was a change in intrahepatic lipid (IHL) concentration. Required sample size was calculated (with IHL as the primary outcome measure) using data from previous meta-analysis [18]. A total of 36 participants (18 per group) would be required to detect a statistically significant change in liver fat following supplementation with an effect size of 0.97, two-sided significance level (α) or 0.05 and 80% power (β = 0.8). Allowing for 30% drop-out rate, we aimed to enroll 25 participants per group. Secondary outcome measures included abdominal VAT and SAT volumes, serum aminotransferases (ALT, AST, GGT), and TG. We also measured changes in anthropometric variables (body weight, BMI, waist circumference) as potential confounders.

An intention-to-treat analysis was employed with group mean changes scores imputed for drop-outs [37]. Sub-analysis of those with NAFLD (IHL ≥ 5.5%) was performed for primary and secondary outcomes. Differences between groups for baseline measures were analyzed using independent sample t-tests for continuous variables and Chi-square test for categorical variables. All outcome measures were assessed by two-way repeated measures analysis of variance (ANOVA) for assessment of time and group × time effects (Statistical Package for the Social Sciences, Release 17.0, SPSS Inc., Chicago, IL, USA), with Bonferroni post-hoc comparison to locate significant differences. Effect size was calculated using Hedges g corrected for bias with 95% confidence intervals. Relationships between change in IHL with intervention and change in potential confounders (diet or physical activity) during treatment, and change in secondary outcome variables were determined by Pearson’s r correlation coefficients. The strength of correlation coefficients was interpreted on the basis of the following definition: weak (*r* < 0.5), moderate (*r* = 0.5–0.69) and strong (*r* ≥ 0.70). Statistical significance was accepted at *p* < 0.05. Values are reported as mean ± standard error (SE).

While the data were being analyzed, a paper was published [38] that indicated the degree of erythrocyte enrichment with DHA and/or EPA may explain why some individuals experience health benefits from supplementation. We therefore decided to also include a brief visual exploration of our data to test this hypothesis in our study cohort.

## 3. Results

### 3.1. Recruitment and Participation

Volunteers (*n* = 222) were screened for eligibility via telephone with 75 of those interested in participating classed as eligible based on the telephone screening questionnaire (supplementary Figure S1). Of these, 54 men who attended the baseline visit were eligible to be randomized. Reasons for ineligibility included waist circumference < 94 cm (*n* = 18), BMI < 25 kg/m^2^ (*n* = 1), and biochemistry indicative of disease (TG > 4.4 mmol/L, *n* = 1; eGFR < 60, *n* = 1). A further four volunteers revoked their consent to participate prior to being randomized. The reasons given included inability to attend MRI visits due to scheduling (*n* = 3) and religious concerns about the sourcing of the fish oil (*n* = 1).

**Figure 1:**
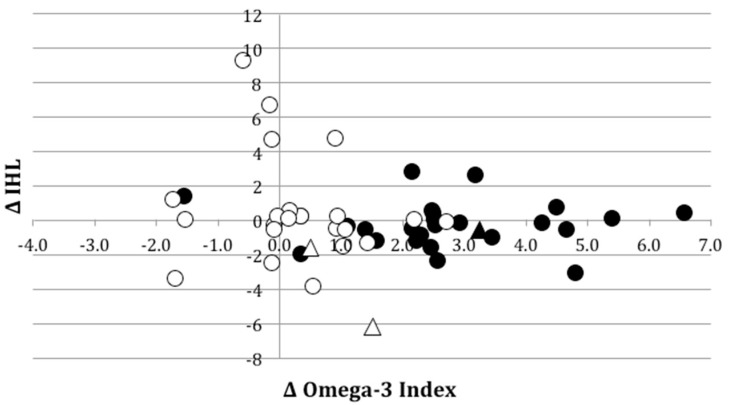
Absolute change in Omega-3 Index vs. absolute change in intrahepatic lipid (IHL) concentration following 12 weeks of supplementation with 1728 mg fish oil (588 mg EPA + 412 mg DHA) versus placebo. Solid circles: Fish Oil group, individuals with <5% weight change during intervention; unfilled circles: Placebo group, individuals with <5% weight change; triangles: individuals with weight change ≥5% (filled: Fish Oil; unfilled: Placebo).

In total, 50 participants were randomized, began supplementation and were analyzed for primary and secondary outcomes. Two participants in the Placebo group dropped out before week 3; reasons given included moving to another city (*n* = 1), and adverse events (gastrointestinal discomfort and diarrhea) experienced during the first week of supplementation (*n* = 1). Therefore 48 participants completed the 12-week supplementation, however three participants (Fish Oil *n* = 1, Placebo *n* = 2) were non-compliant to therapy based on capsule count (<80% capsules taken); compliance to Fish Oil treatment was confirmed by observing an increase in erythrocyte EPA and DHA for 23 out of the 25 (92%) participants who completed the Fish Oil intervention (Figure 1). When questioned, 48/50 participants reported being unaware of the treatment allocation; two participants correctly identified treatment allocation on the basis of suspicion.

### 3.2. Baseline Measures

Baseline characteristics of participants who commenced supplementation (*n* = 50) are shown in Table 1. Although there were no significant differences between groups, IHL tended to be higher in Placebo versus Fish Oil (*p* = 0.07, Table 1). IHL content ranged from 0.6 to 18.2%, and 16 participants (32%) were classified as having NAFLD (Fish Oil: *n* = 6, Placebo: *n* = 10). Participants were similar for all other outcome measures (*p* > 0.05) except TG, where, although still within the normal reference range, the Placebo group has higher baseline levels (Table 1).

### 3.3. Effects of 12 Weeks of Supplementation

The effects of the 12-week intervention are summarized below and in Table 2. The raw mean and SE values for outcomes measured at week 6 and 12 are provided in supplementary Table S1.

#### 3.3.1. Liver Fat Concentration and Body Composition

There was no significant change in IHL content, VAT, SAT, or any anthropometric outcome measure (waist circumference, percent body fat) in either group over the 12-week intervention. Mean body weight of the study cohort remained stable over 12 weeks, however three individuals in the Placebo changed weight (gained or lost) by ≥5% from baseline.

**Figure 2:**
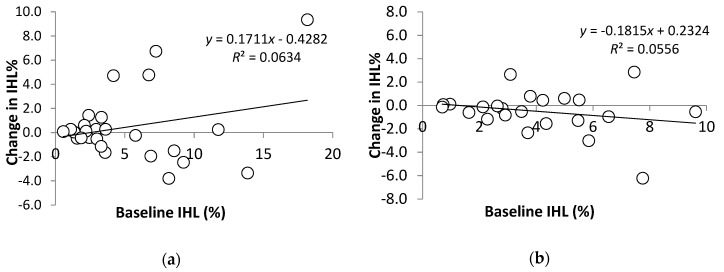
Change in IHL% with respect to baseline IHL% following 12 weeks of supplementation, stratified by enrichment of erythrocyte EPA and DHA: (**a**) Individuals with <2% increase in DHA and <0.7% increase in EPA; (**b**) ≥2% increase in DHA and/or ≥0.7% increase in EPA.

There was no significant group × time interaction for Fish Oil versus Placebo on liver fat (*p* = 0.714). There was a significant positive correlation between relative change in IHL% and change in body weight (*r* = 0.498, *p* < 0.001), and change in IHL% and change in VAT (*r* = 0.294, *p* = 0.038) over 12 weeks. There was no correlation between relative change in IHL% and changes in Omega-3 Index (Figure 1), TG or serum aminotransferases (*p* > 0.05 for all). However, the relationship between baseline IHL% and change in IHL% was modified by the magnitude of EPA+DHA enrichment of erythrocytes (Figure 2).

**Figure 3:**
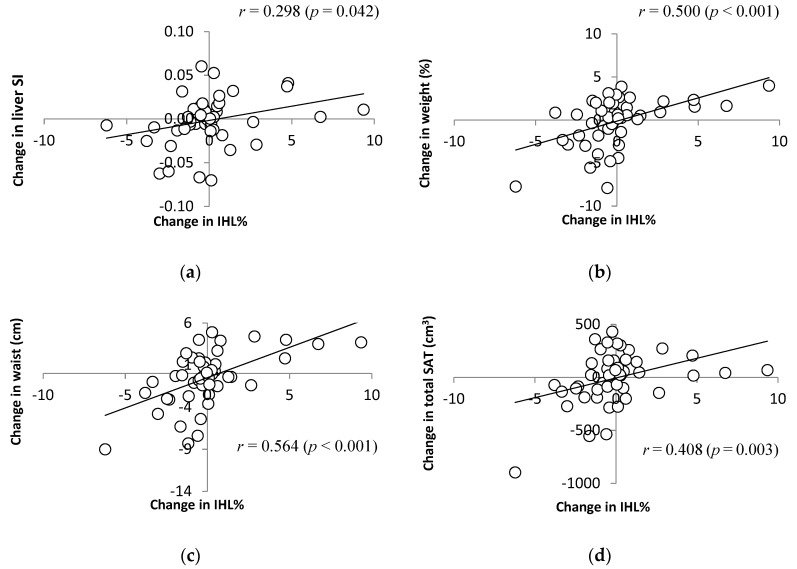
Change in intrahepatic lipid concentration (IHL%) with respect to change in (**a**) liver saturation index (SI); (**b**) weight (% change from original body weight); (**c**) waist (cm); and (**d**) total subcutaneous adipose tissue (SAT; cm^3^) following 12 weeks of supplementation with 2 g Fish Oil or Olive Oil per day. Note: this figure displays change in IHL regardless of supplement group.

Absolute change in IHL% was weakly correlated with changes in hepatic saturation index (i.e., the proportion of liver fat that is saturated) (*r* = 0.298, *p* = 0.042), moderately correlated with changes in weight and waist circumference (*r* = 0.500, *p* < 0.001; *r* = 0.564, *p* < 0.001, respectively), and weakly correlated with change in total SAT volume (*r* = 0.408, *p* = 0.003) (Figure 3), and change in IHL% and SAT at umbilicus (*r* = 0.350, *p* = 0.010). Regression analysis including baseline IHL%, change in body weight, and treatment group revealed that change in body weight was the strongest predictor of absolute change in liver fat (β = 0.489, *p* < 0.001), whereas relative change in IHL% was predicted by baseline liver fat (β = 0.829, *p* < 0.001) and change in body weight (β = 0.262, *p* < 0.001), but not treatment group (β = −0.006, *p* = 0.935).

#### 3.3.2. Blood Biochemistry

Mean fasting serum aminotransferases did not exhibit a group × time interaction over 12 weeks. There was no effect of fish oil supplementation on blood lipids, or any biochemical parameters in this cohort (Table 2), however all biochemistry measures remained within the “normal” range at all time points. Repeated measures ANOVA found no significant differences across time or between groups for erythrocyte saturated fatty acids (SFA), erythrocyte monounsaturated fatty acids (MUFA) or total erythrocyte PUFA (*p* > 0.05 for all). However, there was a statistically significant reduction in erythrocyte omega-6 PUFA in the Fish Oil group that mirrored the increase in erythrocyte omega-3 PUFA also observed in this group across both the 6- and 12-week time points (*p* < 0.001 for both); these differences were not seen in the Placebo group, where the ratio of erythrocyte omega-6 to omega-3 remained stable throughout the 12-week supplementation period.

#### 3.3.3. Sub-Analysis of Participants with NAFLD

Sub-analysis of participants with NAFLD (IHL > 5.5%, *n* = 16) revealed minimal differences between treatments (relevant data presented in text, not tables/figures). A group × time effect was observed for AST (*p* = 0.012) where the Placebo group decreased slightly (by 2.9 U/L) and the Fish Oil group increased slightly (5.7 U/L), with both groups remaining within the normal range for AST. Group × time effects were also observed for body fat% by BIA (*p* = 0.019), erythrocyte fatty acid composition (EPA *p* = 0.001), DHA *p* = 0.046, total omega-3 PUFA *p* = 0.002), and a time effect was observed for erythrocyte fatty acid composition (EPA *p* = 0.001, DHA *p* = 0.004, total omega-3 PUFA *p* < 0.001). A group effect was observed for weight (*p* = 0.033). There was no difference in liver fat between groups at any time point throughout the study, nor was there an overall change in liver fat over the course of the study.

#### 3.3.4. Self-Reported Habitual Dietary Intake and Physical Activity

Habitual diet and physical activity were not significantly different at baseline or during the intervention (*p* > 0.05 for all, supplementary Table S2), except for percent intake of fat as MUFA, where the Placebo group tended to have a slightly decreased, and the Fish Oil group an increased, relative intake of MUFA at 12 weeks (group × time interaction: *p* = 0.018), however there was no overall increase in fat intake, and it appears that increased MUFA intake was not as a substitute for one specific type of fat as no significant changes were observed in saturated or polyunsaturated fat intake (supplementary Table S2). The cohort as a whole reported a small decrease in consumption of sugars over the 12-week intervention (11.5 ± 6.8 g/day reduction from baseline, *p* = 0.049), however given the small size of this change, and the lack of change in overall carbohydrate intake and total energy intake, this is not expected to be clinically significant.

## 4. Discussion

In this study, using gold-standard non-invasive techniques for the assessment of IHL concentration and composition, and an omega-3 dose that has previously been shown to result in reductions in liver fat [18], we failed to observe a hepatic benefit. Similarly, we found no effect of fish oil supplementation on serum aminotransferase levels. Finally, no change was observed in the volume of abdominal visceral or subcutaneous adipose tissue compartments after supplementation.

The finding of no effect of fish oil on hepatic steatosis is opposite to the results of the majority of available trials [18]. However it should be noted that many of these studies recruited for the presence of NAFLD, and therefore mean liver fat levels were higher than the present cohort. Sub-analysis in the current study of those with NAFLD did not show a benefit of omega-3 supplementation on liver fat, and while sample size was calculated in order to achieve sufficient power to detect a significant benefit of omega-3 PUFA supplementation on liver fat if it existed, the relatively lower mean liver fat level in the present sample could yet have resulted in a type 2 error. Furthermore, the majority of the studies in adults available to date generally showed a reduction in liver fat inferred by ultrasound [18], and whilst these findings may be of clinical significance [39], unlike ^1^H-MRS, ultrasound does not enable quantitative assessment of liver fat concentration to a level appropriate to detect the small change often seen in intervention studies [40]. Vega et al. [40] also observed no effect of omega-3 PUFA supplementation on liver fat measured by ^1^H-MRS, and similarly the hepatic response to supplementation was highly variable even in participants with NAFLD [40]. Recent studies in NASH showed opposing effects of higher doses of omega-3 PUFA on MRI- or biopsy-assessed liver fat [41,42,43,44], however two studies [38,44] that did not observe an overall effect of ≥12 months omega-3 supplementation on liver fat showed independent effects of DHA and EPA on liver fat [38].

The mechanisms by which omega-3 PUFA is expected to reduce liver fat levels include downregulation of transcription factors such as sterol regulatory element-binding protein-1 (SREBP-1) and carbohydrate response element-binding protein (ChREBP), thereby lowering hepatic *de novo* lipogenesis [45], altering the balance between hepatic lipid oxidation and production, favoring oxidation, and hence lowering hepatic fat levels. Anti-inflammatory effects of omega-3 PUFA may also reduce pro-inflammatory cytokines and oxidative stress in adipocytes, leading to a lower rate of ectopic lipid accumulation in the liver [45]. Reduction in *de novo* lipogenesis also results in a concomitant reduction in TG-rich very-low-density lipoprotein (VLDL) release into the blood, effectively lowering blood TG levels [45]. The blood TG-lowering effect of omega-3 PUFA supplementation may also be mediated by enhanced activity of adipose tissue lipoprotein lipase [46], however this may require higher doses (>2 g EPA+DHA/day) than used in the current study to effectively lower blood TG [46,47]. Discussion continues as to the minimum effective dose required for hepatic lipid lowering using omega-3 PUFA [45].

To date, studies in NASH cohorts have tended to use higher doses of omega-3 PUFA (>1800 mg EPA and >1400 mg DHA per day) than the current study [42,48] although not all have done so [43]. Studies in NAFLD have used a wider variety of doses of EPA and DHA, ranging from 375 mg EPA and 625 mg DHA per day [49] to 4630 mg EPA and 2150 mg DHA per day [40]. The current study used a dose at the lower end of the spectrum of published studies, however smaller doses have been shown to be effective in reducing liver fat in adults with NAFLD [50]. In the current study, a higher dose of EPA was given than DHA. The contribution of EPA and DHA to the supplements in published studies has no clear pattern, with some studies using more EPA than DHA [38,40,41,48,50] and others using more DHA than EPA, or the same quantity of each [42,43,49,51]. Together with the present study, this raises some uncertainty over the effectiveness of omega-3 PUFA as a general agent for liver fat prevention or reduction in all individuals, and that unless very high doses of omega-3 PUFA are used, perhaps individuals with higher liver fat may benefit from supplementation over those with lower liver fat. However, it is noteworthy that even in studies reporting a mean benefit, only between 35 and 65% of participants with diagnosed NAFLD experienced a reduction in steatosis with omega-3 PUFA supplementation [49,52,53,54,55].

Outside influences such as changes in diet and/or physical activity or even small changes in body weight appear to significantly influence the hepatic benefit [7,41]. Although no effect of diet/physical activity was observed in our study, it is noteworthy that change in body weight and VAT (albeit small and non-significant at a group level), and not supplementation status, accounted for most of the variance in liver fat change at an individual level. In addition, although expected not to benefit participants except for protecting the highly unsaturated fatty acids from oxidation prior to consumption, the inclusion of the coenzyme Q10 in the fish oil capsule may have had an independent effect in the Fish Oil group, although the overall results suggest otherwise.

Strengths of this study include the quantification of liver fat using ^1^H-MRS and measurement of omega-3 incorporation in the body via change in Omega-3 Index. However, it is possible that given the low mean IHL% at baseline, a type II error may have occurred. Furthermore, individuals with low habitual omega-3 PUFA intake may experience the greatest benefit from supplementation [56]. If this is the case, it is possible that our study cohort did not exhibit an overall benefit from supplementation because individuals may have had a sufficient or high habitual intake of omega-3 PUFA prior to the intervention. Indeed, although the dietary software used to analyze habitual intake was unable to calculate intake of omega-3 PUFA (only total PUFA intake), baseline Omega-3 Index was generally above the suggested threshold for optimum protection (>8.0%) for most study participants [29], and relatively high compared with several other studies [57] (for example, mean ± SE: 5.1 ± 0.1% in overweight/obese Australian men [58], compared with 7.9 ± 0.2% in the present study). Omega-3 PUFA intake and status have previously been linked to a reduction in chronic disease risk [29,59], partly through a benefit in blood lipid levels [60]. In contrast to a number of meta-analyses [60,61,62] and similar studies in NAFLD [49,50,53,55], in the present study omega-3 PUFA supplementation did not lower blood TG. This is not unexpected as the dose of omega-3 PUFA used in this study, while consistent with national dietary guidelines [28,63] and supported by previous NAFLD research [50], was lower than that recommended form reducing TG [59,63].

In conclusion, using gold standard measurement techniques and a randomized placebo-controlled design, this study showed that 12 weeks of omega-3 PUFA supplementation with a commonly employed dosage (just under 2 g fish oil per day) did not alter liver fat, aminotransferases, or visceral adiposity in overweight men. This suggests that, while omega-3 PUFA at this dose may be effective at reducing liver fat in those with overt NAFLD, 1000 mg EPA+DHA per day may not be sufficient to recommend for the sole purpose of reducing hepatic and/or visceral fat in men who, while overweight and at increased risk of NAFLD, are otherwise apparently healthy. Nevertheless, the factors determining the efficacy of omega-3 PUFA supplementation on an individual level need to be clarified.

## Figures and Tables

**Table 1:** Baseline participant characteristics.

Characteristic	Total (*n* = 50)	Placebo (*n* = 25)	Fish Oil (*n* = 25)	*p*-Value ^a^
Age (years)	34.2 (1.5)	34.7 (2.3)	33.6 (2.0)	0.723
Caucasian ethnicity (*n*, %)	44 (86)	23 (92)	21 (84)	0.384
**Anthropometry**				
Weight (kg)	90.9 (1.3)	92.4 (2.0)	89.3 (1.6)	0.221
Waist circumference (cm)	100.5 (0.7)	101.0 (0.9)	99.9 (1.1)	0.435
BMI (kg·m^−2^)	27.9 (0.2)	28.0 (0.3)	27.8 (0.3)	0.646
**Liver fat and adiposity**				
IHL (%)	4.6 (0.5)	5.5 (0.9)	3.7 (0.4)	0.078
Hepatic SI	0.949 (0.006)	0.950 (0.006)	0.948 (0.010)	0.830
SAT at umbilicus (cm^3^)	279.7 (9.1)	285.7 (12.4)	273.6 (13.4)	0.510
VAT at umbilicus (cm^3^)	103.5 (8.0)	104.1 (12.4)	102.9 (10.5)	0.942
Body Fat by BIA (%)	23.0 (0.4)	22.8 (0.6)	23.1 (0.5)	0.660
NAFLD (Y/N)	16/34	10/15	6/19	0.225
**Diet and exercise**				
Reported habitual energy expenditure (KJ/day) ^b^	16,590 (561)	17,569 (998)	15,630 (489)	0.084
Reported energy intake (kJ/day)	10,529 (416)	10,619 (652)	10,436 (531)	0.823
**Liver function tests**				
ALT (U/L)	34.4 (1.7)	34.5 (2.4)	34.3 (2.4)	0.962
AST (U/L)	28.6 (1.3)	26.5 (0.9)	30.7 (2.4)	0.106
GGT (U/L)	30.3 (2.4)	30.5 (3.2)	30.2 (3.6)	0.947
**Other biochemistry**				
Triglycerides (mmol/L)	1.3 (0.1)	1.5 (0.1)	1.0 (0.1)	0.001
Omega-3 Index ^c^	7.9 (0.2)	8.0 (0.3)	7.9 (0.3)	0.821

Data presented as mean (SE). ^a^ *p*-value for between group comparisons. ^b^ Habitual energy expenditure measured by Bouchard questionnaire. ^c^ Omega-3 Index: erythrocyte EPA+DHA as % of total membrane fatty acids. Abbreviations: ALT, alanine aminotransferase; AST, aspartate aminotransferase; BIA, bioelectrical impedance analysis; BMI, body mass index; GGT, gamma-glutamyl aminotransferase; IHL, intrahepatic lipid; NAFLD, non-alcoholic fatty liver disease; SAT, abdominal subcutaneous adipose tissue; SI, saturation index; VAT, visceral adipose tissue. Laboratory reference ranges: ALT 5–55 U/L; AST 5–55 U/L; GGT ≤ 60 U/L; Triglycerides ≤ 2.5 mmol/L.

**Table 2:** Changes in anthropometric, hepatic, abdominal fat, and biochemical outcomes during 12 weeks of supplementation with Fish Oil or Placebo.

	Placebo		Fish Oil		P (*t*)	P (*g* × *t*) ^1^
	Week 0 to 6	Week 0 to 12	Week 0 to 6	Week 0 to 12		
**Anthropometry**						
Weight (kg)	0.2 (0.4)	0.3 (0.5)	−0.2 (0.2)	−0.4 (0.4)	0.856	0.517
Waist (cm)	0.0 (0.6)	−0.1 (0.7)	−0.3 (0.3)	−0.9 (0.6)	0.385	0.439
BMI (kg·m^-2^)	0.0 (0.1)	0.1 (0.2)	−0.1 (0.1)	−0.1 (0.1)	0.869	0.475
**Liver fat and adiposity**						
IHL (%)	0.7 (0.7)	0.3 (0.7)	0.0 (0.3)	−0.2 (0.3)	0.157	0.801
Hepatic SI	0.000 (0.008)	0.002 (0.006)	−0.009 (0.007)	−0.006 (0.005)	0.514	0.884
SAT, umbilicus (cm^3^)	1.6 (4.7)	0.1 (5.4)	−3.0 (3.2)	−2.7 (2.8)	0.823	0.746
VAT, umbilicus (cm^3^)	−1.4 (5.5)	−3.8 (4.6)	−6.1 (3.6)	−1.3 (4.7)	0.683	0.197
SAT, total (L)	0.00 (0.09)	0.00 (0.12)	−0.07 (0.05)	−0.07 (0.07)	0.909	0.970
VAT, total (L)	0.00 (0.07)	−0.07 (0.07)	−0.12 (0.07)	−0.07 (0.09)	0.768	0.224
Body fat, BIA (%)	0.1 (0.2)	0.0 (0.3)	−0.5 (0.3)	−0.2 (0.4)	0.525	0.273
**Liver function tests**						
ALT (U/L)	−2.0 (1.5)	−3.6 (1.1)	2.0 (2.5)	4.1 (3.6)	0.894	0.387
AST (U/L)	−0.8 (0.7)	−2.3 (0.6)	0.6 (3.2)	−0.8 (3.1)	0.464	0.983
GGT (U/L)	−0.1 (1.4)	−1.3 (1.4)	0.0 (2.1)	2.0 (1.8)	0.596	0.052
**Other biochemistry**						
Triglycerides (mmol/L)	0.0 (0.12)	0.0 (0.13)	0.1 (0.08)	0.1 (0.08)	0.772	0.694
Omega-3 Index	0.4 (0.26)	0.3 (0.21)	1.8 (0.27)	2.8 (0.34)	0.018	0.006

Data are presented as mean change (SE). ^1^ *p*-values for time (*t*), and group × time (*g* × *t*) interactions, statistically significant results in bold. Abbreviations: ALT, alanine aminotransferase; AST, aspartate aminotransferase; BIA, bioelectrical impedance analysis; BMI, body mass index; GGT, gamma-glutamyl aminotransferase; IHL, intrahepatic lipid; SAT, abdominal subcutaneous adipose tissue; SI, saturation index; VAT, visceral adipose tissue. See Table 1 for baseline values and laboratory reference ranges.

## Supplementary Materials

The following are available online at https://www.mdpi.com/2072-6643/11/2/475/s1, Figure S1: Flow diagram of volunteer recruitment and study participation, Table S1: Anthropometric, hepatic, abdominal fat and biochemical outcomes during 12 weeks of supplementation with Fish Oil or Placebo, Table S2: Self-reported dietary intake and energy expenditure.
