# Supplementary material for: Effect of Fish Oil Supplementation on Hepatic and Visceral Fat in Overweight Men: A Randomized Controlled Trial
Source: Nutrients. 2019 Feb 23;11(2):475. doi: 10.3390/nu11020475 (PMC6413081; doi:10.3390/nu11020475)
Supplement: Supplementary file 1 [file nutrients-11-00475-s001.pdf]

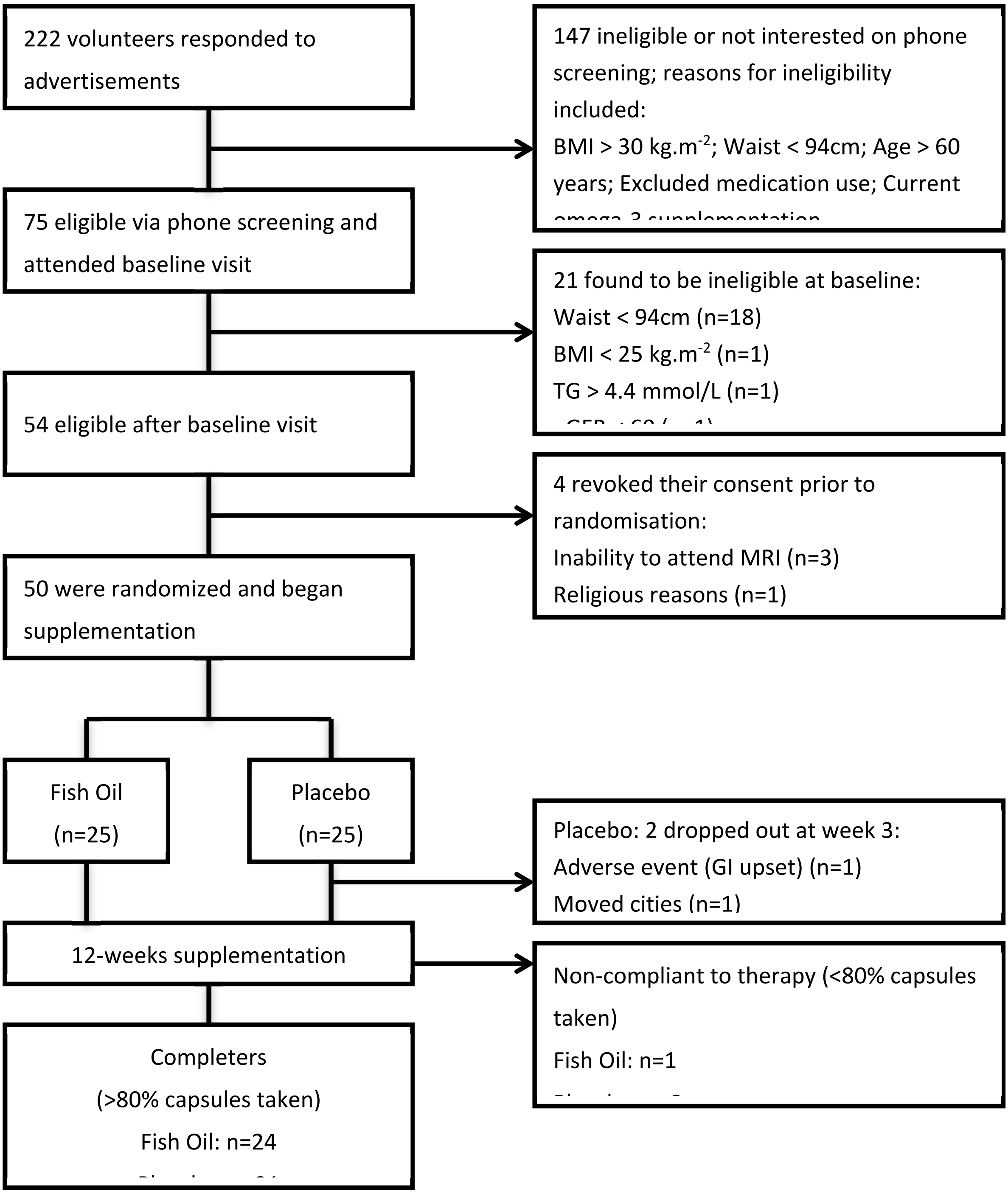

Figure S1. Flow diagram of volunteer recruitment and study participation. Final analyses were conducted as intention to treat, and included all participants who initiated supplementation regardless of compliance to therapy. Group means imputed for drop-outs and missing data (see Methods for details).

Table S1. Anthropometric, hepatic, abdominal fat and biochemical outcomes during 12 weeks supplementation with Fish Oil or Placebo.

	**Placebo**		**Fish Oil**		**P (*t*)**	**P (*g*)**	**P (*g* × *t*)**[1]
	**Week 6**	**Week 12**	**Week 6**	**Week 12**			
Anthropometry							
Weight (kg)	92.6 (2.1)	92.7 (2.1)	89.1 (1.6)	88.9 (1.7)	0.936	0.185	0.405
Waist (cm)	101.3 (1.1)	100.9 (1.2)	99.6 (1.2)	99.0 (1.3)	0.424	0.343	0.527
BMI ($kg.m^2$)	28.0 (0.3)	28.0 (0.3)	27.7 (0.3)	27.6 (0.3)	0.870	0.489	0.425
Liver fat and adiposity							
IHL (%)	6.2 (1.2)	5.8 (1.2)	3.8 (0.5)	3.5 (0.5)	0.475	0.061	0.542
Hepatic SI	0.950 (0.006)	0.953 (0.005)	0.938 (0.010)	0.942 (0.009)	0.610	0.406	0.594
SAT, umbilicus (cm^3)	287.3 (12.3)	285.8 (13.1)	270.6 (13.2)	270.9 (13.5)	0.900	0.423	0.717
VAT, umbilicus (cm^3)	102.7 (12.5)	100.2 (11.3)	96.8 (10.1)	101.6 (9.9)	0.476	0.901	0.503
SAT, total (L)	6.24 (0.28)	6.24 (0.29)	6.00 (0.26)	6.00 (0.26)	0.854	0.722	0.672
VAT, total (L)	3.34 (0.31)	3.28 (0.30)	3.04 (0.26)	3.08 (0.28)	0.359	0.729	0.474
Body fat, BIA (%)	22.9 (0.6)	22.8 (0.7)	22.6 (0.5)	22.9 (0.6)	0.528	0.921	0.349
Liver function tests							
ALT (U/L)	32.4 (2.3)	30.9 (2.1)	36.4 (2.7)	38.4 (4.2)	0.975	0.262	0.119
AST (U/L)	25.7 (1.0)	24.3 (0.8)	31.3 (3.3)	29.9 (1.7)	0.611	0.021	0.899
GGT (U/L)	30.3 (2.8)	29.2 (2.8)	30.1 (3.4)	32.2 (3.9)	0.880	0.962	0.224
Other biochemistry							
Triglycerides (mmol/L)	1.5 (0.1)	1.5 (0.1)	1.2 (0.1)	1.1 (0.1)	0.739	0.006	0.450
Omega-3 Index	8.4 (1.7)	8.3 (1.5)	9.6 (1.7)	10.6 (2.4)	<0.001	0.013	<0.001

Data are presented as mean ± SE. [1] P-values for group (*g*), time (*t*), and group × time (*g* × *t*) interactions. Abbreviations: ALT, alanine aminotransferase; AST, aspartate aminotransferase; BIA, bioelectrical impedance analysis; GGT, gamma-glutamyl aminotransferase; IHL, intrahepatic lipid; SAT, abdominal subcutaneous adipose tissue; SI, saturation index; VAT, visceral adipose tissue. See Table 1 for baseline measures and laboratory reference ranges.

Table S2. Self-reported dietary intake and energy expenditure.

	Placebo			Fish Oil			*P*	*P*
	Week 0	Week 6	Week 12	Week 0	Week 6	Week 12	*(t)*	*(g x t)*
Energy Intake								
Fat (g/day)	90.0	89.2	91.7	96.0	90.2	89.8	0.771	0.697
	(7.3)	(5.3)	(4.3)	(7.0)	(5.2)	(6.6)		
% Energy intake	30.3	32.1	33.1	33.0	32.8	32.9	0.338	0.232
	(1.1)	(1.1)	(1.1)	(1.2)	(1.0)	(1.2)		
- SFA (g/day)	34.2	35.1	36.6	39.0	35.9	34.2	0.812	0.227
	(3.1)	(2.6)	(2.1)	(3.6)	(2.6)	(3.1)		
- SFA (%fat intake)	41.0	42.3	43.7	43.0	42.9	41.1	0.822	0.065
	(1.3)	(1.5)	(1.8)	(1.5)	(1.5)	(1.4)		
- MUFA (g/day)	34.6	33.4	34.0	35.5	34.2	36.3	0.771	0.916
	(3.3)	(2.2)	(2.1)	(2.6)	(2.3)	(2.9)		
- MUFA (%fat intake)	40.5	39.8	38.9	38.9	40.2	42.6	0.496	**0.018**
	(1.10	(1.0)	(1.1)	(1.0)	(1.1)	(1.0)		
- PUFA (g/day)	14.8	14.4	14.9	14.7	13.7	12.9	0.571	0.554
	(1.4)	(1.4)	(1.3)	(1.1)	(1.0)	(1.0)		
- PUFA (%fat intake)	18.3	17.8	17.4	17.6	16.9	16.3	0.317	0.974
	(1.2)	(1.2)	(1.2)	(1.3)	(1.5)	(1.)		
Carbohydrate (g/day)	287.6	268.6	261.6	267.8	248.1	247.1	0.095	0.959
	(21.1)	(16.3)	(18.3)	(14.5)	(16.5)	(11.7)		
% Energy intake	43.9	42.8	41.4	42.2	40.7	42.4	0.317	0.183
	(1.4)	(1.5)	(1.7)	(1.4)	(1.2)	(1.3)		
- Sugar (g/day)	127.3	104.8	115.0	112.2	101.9	101.6	**0.049**	0.623
	(13.1)	(9.6)	(13.7)	(10.2)	(10.9)	(7.8)		
Protein (g/day)	114.8	110.6	108.2	108.2	118.8	104.4	0.434	0.486
	(5.9)	(5.9)	(4.3)	(6.0)	(12.0)	(4.7)		
% Energy intake	19.3	18.8	19.0	18.1	18.8	18.7	0.962	0.611
	(1.0)	(0.9)	(1.0)	(0.7)	(0.7)	(0.7)		
Total energy intake (MJ/day)	10.6	10.1	10.0	10.4	10.0	9.4	0.295	0.876
	(0.7)	(0.5)	(0.5)	(0.5)	(0.5)	(0.4)		
Energy Expenditure								
Self-reported EE (kcal/kg/day)	45.4	43.8	43.2	41.8	41.3	42.0	0.151	0.172
	(1.9)	(1.7)	(1.8)	(0.9)	(0.7)	(1.0)		
Total self-reported EE (MJ/day)	17.6	17.1	16.8	15.6	15.4	15.6	0.164	0.269
	(1.0)	(1.0)	(1.0)	(0.5)	(0.4)	(0.5)		
Total measured EE (MJ/day)	13.5	13.2	13.4	13.3	13.6	13.2	0.781	0.313
	(0.3)	(0.3)	(0.2)	(0.4)	(0.4)	(0.4)		
Steps per day	11068	10395	10180	9581	9094	9438	0.379	0.696
	(839)	(813)	(901)	(582)	(477)	(694)		

Average METs	1.51 (0.03)	1.48 (0.03)	1.52 (0.03)	1.50 (0.03)	1.54 (0.03)	1.51 (0.03)	0.965	0.264
Lying down (hr:min)	8:07 (0:18)	8:19 (0:17)	8:09 (0:15)	7:51 (0:11)	7:49 (0:13)	7:59 (0:12)	0.855	0.676
Sleep (hr:min)	6:30 (0:17)	6:46 (0:14)	6:43 (0:14)	6:33 (0:11)	6:26 (0:13)	6:41 (0:10)	0.554	0.456
Time on body (hr:min)	23:23 (0:07)	23:14 (0:08)	23:13 (0:10)	23:31 (0:06)	23:21 (0:10)	23:13 (0:06)	**0.026**	0.702

Data presented as mean (SE), statistically significant results in **bold**. Abbreviations: *t*, time; *g x t*, group x time; SFA, saturated fatty acids; EE, energy expenditure; METs, metabolic equivalents; MUFA, monounsaturated fatty acids; PUFA, polyunsaturated fatty acids. Energy intake calculated from 3-day estimated food records, using FoodWorks™ software; self-reported energy expenditure calculated from Bouchard activity questionnaire [32]; measured energy expenditure (including steps per day, METs, lying down and sleep) calculated from 3-day activity recording using SenseWear™ triaxial accelerometer.
